# Plasma Fibrin Clot Properties Are Unfavorably Altered in Women following Venous Thromboembolism Associated with Combined Hormonal Contraception

**DOI:** 10.1155/2019/4923535

**Published:** 2019-11-18

**Authors:** Magdalena Piróg, Sławomir Piwowarczyk, Anetta Undas

**Affiliations:** ^1^Gynecological Endocrinology Department, Jagiellonian University Medical College, Krakow, Poland; ^2^Department of Pregnancy Pathology, Ujastek Medical Center, Gynecology and Obstetrics Hospital, Kraków, Poland; ^3^Institute of Cardiology, Jagiellonian University Medical College, and John Paul II Hospital, Krakow, Poland

## Abstract

The use of hormonal contraception is associated with an increased risk of venous thromboembolism (VTE). Unfavorably altered fibrin clot phenotype has been reported in patients following unprovoked VTE who are at risk of recurrences. It remains unknown whether fibrin clot characteristics in women with contraception-related VTE differ from those in unprovoked VTE. We studied three age-matched groups of women: (1) after contraception-related VTE, (*n* = 48) (2) after unprovoked VTE (*n* = 48), and (3) controls (*n* = 48). Plasma fibrin clot permeability (*K*_s_), turbidity of clot formation, efficiency of fibrinolysis using clot lysis time (CLT), and rate of increase in D-dimer during lytic clot degradation (D-D_rate_), along with thrombin generation and fibrinolysis proteins were determined. Compared with the controls, patients following contraception-related and unprovoked VTE formed faster (lag phase, -8.8% and -20.4%, respectively) fibrin clots of increased density (*K*_s__,_ -8.6% and -13.4%, respectively) displaying impaired fibrinolysis as evidenced by prolonged CLT (+11.5% and +14.5%, respectively) and lower D-D_rate_ (-7.1% and -5.6%, respectively), accompanied with higher plasminogen activator inhibitor-1 (PAI-1, +14.9% and +17.8%, respectively) and elevated peak thrombin generation (+63.8% and +36.7%, respectively). The only differences between women with unprovoked and contraception-related VTE were lower fibrin mass in plasma clots (D-D_max_, -8.6%), along with higher peak thrombin generation (+19.8%) and shorter lag phase (-6.8%) in the latter group. This study suggests that women after contraception-related VTE, similar to those following unprovoked VTE, have denser fibrin clot formation and impaired clot lysis. These findings might imply higher risk of VTE recurrence in women with the prothrombotic clot phenotype.

## 1. Introduction

Venous thromboembolism (VTE), including both pulmonary embolism (PE) and deep vein thrombosis (DVT), is a rare complication of combined hormonal contraceptives (CHCs) with the annual incidence of 7-12 events per 10,000 women [1, 2]. The risk of VTE may vary depending on estrogen dose, formula, and types of progestogens [3, 4]. Both hormones lead to a procoagulant risk profile associated with higher plasma D-dimer, tissue plasminogen activator (tPA), and coagulation factors II, VII, and X [5, 6]. Moreover, CHCs diminish levels of the natural anticoagulants such as protein C and antithrombin and may lead to the resistance to activated protein C [4].

The prothrombotic fibrin clot phenotype denotes formation of stiff fibrin clots composed of compact fiber networks displaying decreased porosity, expressed by low clot permeability coefficient (*K*_s_), and resistance to plasmin-mediated lysis reflected by, e.g., prolonged clot lysis time (CLT) [7]. Unfavorably altered fibrin clot properties have been shown to increase the risk of unprovoked VTE and recurrent PE as well as DVT [8–10].

There is a controversy whether the first contraception-related VTE can be classified as either unprovoked VTE, implicating that hormonal therapy is a “weak” risk factor, or provoked VTE with a known risk factor [11, 12]. Importantly, considering the risks and consequences of long-term anticoagulation, it is necessary to determine if women developing VTE on CHCs should be managed as patients with unprovoked or provoked VTE. Current guidelines recommend an anticoagulant therapy for a minimum of 3 months in both provoked and unprovoked VTE [13]. The extended anticoagulant therapy should also be considered in women with contraception-related VTE [14].

Women with contraception-related VTE have been found to display a mild prothrombotic fibrin clot phenotype, reflected by faster formation of dense meshwork (reflected by lower *K*_s_ values) which is relatively resistant to plasmin-induced lysis [4, 7]. Analysis of VTE patients showed that women with both prolonged CLT and hypofibrinolysis resulting from oral contraceptive usage have a 20-fold increase in the VTE risk when compared with individuals with the shorter CLT and without this risk factor [15].

We hypothesized that unfavorably altered clot characteristics occur in women following contraception-related VTE compared with well-matched control and the plasma fibrin clot phenotype is less prothrombotic than in well-matched women after unprovoked VTE.

## 2. Materials and Methods

### 2.1. Participants

In this case-control study, we enrolled 48 consecutive women with a history of the first contraception-related VTE for further work-up in the Center for Coagulation Disorders in Kraków, Poland, from July 2014 to June 2016.

Women who took various agents containing ethinylestradiol in combination with levonorgestrel or gestoden when taken orally or in combination with norelgestromin when used transdermally, at the time of VTE diagnosis, were eligible. Women who used contraceptives for indication other than the prevention of pregnancy such as endometriosis, polycystic ovary syndrome, and intermenstrual bleeding were ineligible. All women after VTE occurrence stopped taking hormonal contraception.

Patients were eligible if DVT and/or PE diagnosed on oral or transdermal contraceptives were treated for at least 3 months. Exclusion criteria were age above 50 years, recurrent thrombotic event, ischemic heart disease, valvular heart disease, severe hypertension, diabetes with vascular involvement, and the presence of known VTE risk factors, including recent major surgery with prolonged immobilization or trauma, deficiency of antithrombin, protein C or protein S, antiphospholipid syndrome, acute coronary syndrome or ischemic stroke within the previous 3 months, known malignancy, any chronic inflammatory diseases (e.g., rheumatoid arthritis) or signs of an acute infection, advanced chronic renal disease (estimated glomerular filtration rate (eGFR) <30 mL/min), and international normalized ratio (INR) more than 1.2 at the day of blood draw and pregnancy.

We collected data on risk factors and comorbidities. Smoking was defined as the daily use of 1 or more cigarettes. Family history of VTE was specified as a VTE episode in a first-degree relative documented in medical records including imaging examination. Arterial hypertension was defined as a systolic blood pressure of 140 mmHg or higher or a diastolic blood pressure of 90 mmHg or higher, or taking an antihypertensive medication. Obesity is defined as having a body mass index (BMI) of 30 kg/m^2^ or greater. Diabetes mellitus was defined in accordance with the American Diabetic Association Criteria.

At the same time, we recruited two groups matched for age by frequency (48 women in each group):

1. The unprovoked VTE group of women with no known malignancy, major trauma within 6 preceding weeks or surgery requiring general anesthesia, without prior use of oral contraceptive or hormone replacement therapy, and no pregnancy or delivery within the last 3 months
2. The no-VTE control group comprising women free of documented VTE; subjects with mild arterial hypertension or diabetes mellitus without medication were also eligible

DVT was diagnosed based on symptoms and documented using color duplex sonography (visualization of an intraluminal thrombus either in the calf, popliteal, femoral, or in iliac veins). The diagnosis of PE was based on the presence of characteristic symptoms and positive results of high-resolution spiral computed tomography. All women with VTE were treated with low-molecular-weight heparins (LMWH) at therapeutic doses for 2-10 days followed by non-vitamin K antagonist oral anticoagulants (NOAC).

The Bioethical committee approved the study and all subjects signed written consents.

### 2.2. Laboratory Investigations

Venous blood samples were drawn with minimal stasis using atraumatic venipuncture at 08.00–11.0 AM, after an overnight fast. All measurements were performed in VTE patients after 3 months of anticoagulant therapy since the index event. Patients were drawn >24 hours since the last dose of NOACs. Complete blood count, glucose, creatinine, lipid profiles, and INR were assayed by routine laboratory techniques. High-sensitivity C-reactive protein (hs-CRP) was determined by immunoturbidimetry (Roche Diagnostics GmbH, Mannheim, Germany). Plasma D-dimer was measured with the Innovance D-dimer assay (Siemens, Marburg, Germany). Plasma *α*_2_-antiplasmin and plasminogen were measured by chromogenic assays (STA Stachrom antiplasmin and Stachrom plasminogen, Diagnostica Stago, Asniéres, France). Plasma PAI-1 antigen was measured by an ELISA kit (Hyphen). For evaluation of clot properties and thrombin generation, venous blood samples were mixed with 3.2% trisodium citrate (vol/vol, 9 : 1), then centrifuged at 2000 × *g* for 10 min within 30 minutes of the draw, and stored in aliquots at -80°C until analysis. All measurements were performed by technicians blinded to the origin of the samples. Intra-assay and interassay coefficients of variation were 5-7%.

### 2.3. Thrombin Generation

Calibrated automated thrombography (CAT) (Thrombinoscope BV, Maastricht, Netherlands) were used to assess peak thrombin concentration, using in a 96-well plate fluorometer (Ascent Reader, Thermolab Systems OY, Helsinki, Finland) at 37°C according to the manufacturer's instructions, as previously described [16]. Briefly, platelet-poor plasma (80 *μ*L) was diluted with a tissue factor (TF; 20 *μ*L)-based activator (Diagnostica Stago, Asniéres, France) containing recombinant TF (5 pM L^-1)^, phosphatidylserine/phosphatidylcholine/phosphatidylethanolamine vesicles (4 micromolar) and FluCa solution (Hepes, pH 7.35, 20 *μ*L of 100 nM L^−1^ CaCl2, 60 mg mL^−1^ bovine albumin, and 2.5 mM L^−1^ Z-Gly-Gly-Arg-amidometylcoumarin). The maximum concentration of thrombin formed during the time of registration was described as the thrombin peak, and the area under the curve represented ETP. The peak thrombin level was analyzed twice.

### 2.4. Fibrin Permeation

Fibrin clot permeation was determined using a pressure-driven system as described [17]. Briefly, calcium chloride (20 mmol L^−1^) and human thrombin (Sigma, St. Louis, MO, USA; 1 U mL^−1^) were added to a citrated plasma (120 *μ*L). After incubation in a wet chamber, tubes containing the clots were connected via plastic tubing to a reservoir of a buffer (0.01 M Tris, 0.1 M NaCl, pH 7.4) and its volume flowing through the gels was measured within 60 min.

A permeation coefficient (*K*_s_), which indicates the pore size, was calculated from the equation:

(1) $$\begin{array}{c} K_{\text{s}}=\frac{L\cdot \eta \cdot Q}{t\cdot A\cdot \Delta p}, \end{array}$$

where *Q* is the flow rate in time *t*; *L*, the length of a fibrin gel; *μ*, the viscosity of liquid (in poise); *A*, the cross-sectional area (in cm^2^); Δ*p*, a differential pressure (in dyne cm^−2^); and *t*, the percolating time.

### 2.5. Turbidity Measurements

Clot formation was assessed as previously described [15]. Briefly, plasma-citrated samples were mixed 2 : 1 with a Tris buffer, containing human thrombin (Sigma; 0.6 U/mL) and calcium chloride (50 mM), which initiated polymerization.

Both the lag phase of the turbidity curve, which show the time required for initial protofibril formation, and maximum absorbance at the plateau phase (*Δ*Abs_max_), indicating the number of protofibrils per fiber, were recorded. Absorbance was read at 405 nm.

### 2.6. Lysis Assays

Clot lysis time was assessed using two different methods, as previously described [18]. Briefly, CLT was measured in the essay in which citrated plasma was mixed with calcium chloride (15 mmol L^-1)^, 10,000-diluted human TF (Innovin, Siemens) with a final concentration of phospholipid vesicles (0.6 pM, 12 *μ*M), and rtPA (60 ng/mL; Boehringer Ingelheim, Ingelheim, Germany). The turbidity was measured at 405 nm at 37°C. CLT was defined as the time from the midpoint of the clear-to-maximum-turbid transition, which reflects clot formation, to the midpoint of the maximum-turbid-to-clear transition. To measure clot degradation after its formation and stabilization, fibrin clots, formed as for the permeability evaluation, were perfused with a Tris buffer with 0.2 *μ*mol L^−1^ rtPA (Boehringer Ingelheim). D-dimer levels were measured every 20 min in the effluent using an ELISA kit (American Diagnostica). The experiment was stopped, usually after 80–120 min, when the fibrin gel collapsed under the pressure. The maximum rate of increase in D-dimer levels (D–D_rate_) in the buffer and maximum D-dimer concentrations (D–D_max_) were analyzed [19].

### 2.7. Genotyping

Factor V Leiden (FV Leiden), prothrombin 20210A, factor XIII Val34Leu (FXIII Val34Leu), and *α*-fibrinogen Thr312Ala polymorphisms were determined by the polymerase chain reaction followed by restriction fragment length polymorphism analysis, as previously described [10].

### 2.8. Statistical Analysis

The study was powered to have a 90% chance of detecting a 10% difference in *K*_s_, a key measure of clot properties, using a *p* value of 0.05, based on the values of *K*_s_ from a published article [20]. To demonstrate such a difference, or a greater one, 30 patients or more were required in each group.

All calculations were done with STATISTICA 12.0 software (StatSoft, Poland).

Categorical variables are presented as numbers and percentages. Continuous variables are expressed as mean ± standard deviation or median and interquartile range (IQR), as appropriate. The Shapiro–Wilk test was used to assess normality and Levene's test was taken to check equality of variances. Differences between groups were compared using either the Welch's *t*-test or the Mann–Whitney *U* test, depending on the equality of variances for normally distributed variables. Categorical variables were analyzed using either the chi^2^ test or Fisher's exact test. We used Kruskal-Wallis ANOVA with *post hoc* Tuckey test to investigate intergroup differences between multiple groups. Pearson's correlation coefficient (Pearson's *r*) or Spearman's rank correlation coefficient were calculated to assess the linear correlations between variables with a normal or nonnormal distribution, respectively. Associations between the variables were expressed as odds ratios with 95% confidence intervals. Two-sided *p* values <0.05 were considered statistically significant.

## 3. Results

### 3.1. Participants' Characteristics

As shown in Table 1, there were no intergroup differences regarding demographic variables, smoking status, family history of VTE, obesity, and diabetes mellitus (Table 1). In the contraception-related VTE group, the prevalence of arterial hypertension was lower than in both unprovoked VTE and control groups. In terms of routine laboratory tests, compared with the control group, women following unprovoked VTE had slightly higher triglycerides (TG) and increased low-density lipoprotein cholesterol (LDL-C). Other laboratory investigations, including fibrinogen, were similar in the 3 groups (Table 1).

Analysis of the genetic polymorphisms revealed similar carrier frequencies in all groups, except for a higher prevalence of FXIII 34Leu allele carriers in the contraception-related VTE group, compared with the two remaining groups (Table 1). Factor V Leiden mutation tended to occur more often in the contraception-related VTE group than in the unprovoked group (Table 1); however, the difference did not reach the level of statistical significance.

### 3.2. Thrombotic and Fibrinolysis Markers

**Figure 1:**
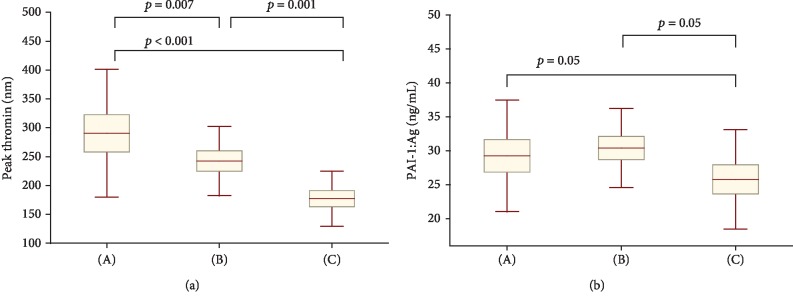
Comparison of peak thrombin (a) and plasminogen activator inhibitor-1 (PAI-1, (b)) in 3 studied groups; ((A) the contraception-related group, (B) the unprovoked VTE group, and (C) the control group).

Peak thrombin generation was higher both in the contraception-related VTE group (+63.8%) and in the unprovoked VTE group (+36.7%) contrary to in the control group (Figure 1(a)). Moreover, women with contraception-related VTE had 19.8% higher peak thrombin generation when compared with unprovoked VTE. No intergroup differences in plasminogen and *α*_2_-antiplasmin were observed (Table 1). Plasma PAI-1 was higher by 14.9% in the contraception-related VTE group and by 17.8% in the unprovoked VTE group when compared with the control group (Figure 1(b)).

### 3.3. Fibrin Clot Properties

**Figure 2:**
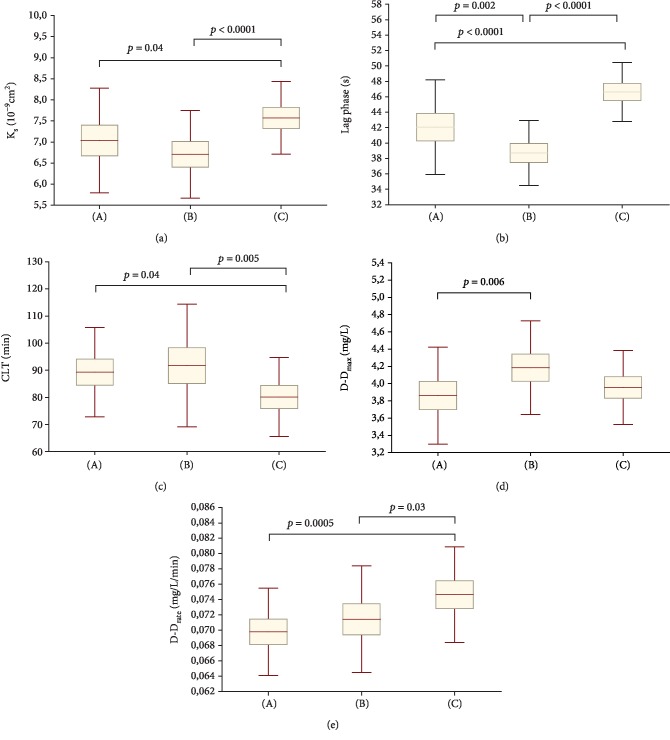
Comparison of fibrin clot permeability coefficient (*K*_s_, (a)), lag phase (b), clot lysis time (CLT, (c)), maximum D-dimer levels in the lysis assay (D-D_max_, (d)), and maximum rate of increase in D-dimer levels in the lysis assay (D-D_rate_, (e)) in the 3 studied groups. Data are shown as mean and standard deviation (SD) ((A) the contraception-related group, (B) the unprovoked VTE group, and (c) the control group).

Women from both contraception-related and unprovoked VTE group had lower *K*_s_ when compared with the control group (8.6% and 13.4%, respectively; Figure 2(a)) indicating the formation of more compact fibrin networks. There was no association between *K*_s_ and thrombin generation in either group.

Both contraception-related and unprovoked VTE groups were characterized by shorter lag phase when compared with the controls (by -8.8% and -20.4%, respectively). Contrary to the unprovoked VTE group, women with contraception-related VTE had longer lag phase (by +8.6%). No intergroup differences in *Δ*Abs_max_ were observed.

Regarding fibrinolysis, both contraception-related and unprovoked VTE groups showed longer CLT when compared with the control group (by 11.5% and 14.5%, respectively; Figure 2(c)). CLT was positively correlated with PAI-1 Ag in both contraception-related and unprovoked groups (*r* = 0.63, *p* = 0.005 and *r* = 0.68, *p* = 0.007, respectively). CLT tended to be positively associated with plasminogen (*r* = 0.74, *p* = 0.064 and *r* = 0.83, *p* = 0.072, respectively) and antiplasmin (*r* = 0.51, *p* = 0.061 and *r* = 0.48, *p* = 0.070, respectively).

As shown in Figure 2(d), the D-D_max_ was lower by 8.6% in the contraception-related VTE group when compared with the unprovoked VTE group. Contrary to the control group, both contraception-related and unprovoked VTE groups were characterized by slightly lower D-D_rate_ (by 7.1% and 5.6%, respectively, Figure 2(e)). Regarding types of contraception, we observed 13.2% lower D-D_rate_ (*post hoc* Tuckey *p* = 0.006) and 9.3% higher D-D_max_ (*post hoc* Tuckey *p* = 0.047) in women with VTE provoked by oral contraceptives with 2^nd^-generation progesteron when compared with transdermal contraception-provoked VTE (Table 2). No other differences in the parameters measured were observed in relation to the type of contraception used before VTE event (Table 2).

## 4. Discussion

The current study shows that women who experienced the first contraception-related episode of VTE, compared with controls, demonstrate increased thrombin generation and prothrombotic plasma fibrin clot phenotype, evidenced by faster formation of denser fiber networks and reduced clot susceptibility to lysis. We found no differences in most prothrombotic variables between women following VTE related to hormonal contraception and those after unprovoked VTE, apart from slightly lower maximum D-dimer levels measured during lytic clot degradation and higher peak thrombin generation and longer lag phase in the first group. Our study suggests that women with both contraception-related and unprovoked VTE may remain at a similarly increased risk of a subsequent thrombotic event, which might imply benefits from longer anticoagulation in a subset of those patients.

There is a controversy whether contraception-related first VTE can be classified as either unprovoked VTE, thus regarding hormonal therapy as a “weak” risk factor, or provoked VTE [11, 12]. It has been reported that in young women with a first episode of VTE, no significant association was found between exposure to CHCs and the incidence of recurrent VTE after adjustment for age or after restricting the analysis to major unprovoked VTE: incidence rate of recurrence 17.9/1,000/year (95% confidence interval (CI): 9.6-33.2) in women with CHC as compared with 17.6/1,000/year (95% CI: 6.6-47) with an incidence ratio of 0.7 (95% CI: 0.2-2.4, *p* = 0.59) [21–25]. Other studies reported that the risk of VTE recurrence after anticoagulant therapy for the first unprovoked VTE episode is comparable between women on or off estrogen containing CHCs and HRT [24, 26]. It has been reported that the risk of recurrent VTE is similar in estrogen users at the time of their first VTE episode (9.7 per 1,000 patient-years; 95% CI: 4.3–21.5) and nonusers (16.2; 95% CI: 8.7–30.2) [27]. Moreover, other study showed an incidence rate for recurrent VTE of 5.1% for the first year, 7.8% for the first 2 years, 14.2% for the first 5 years, and 28.6% for 10 years in CHCs users, which is a high risk of recurrence given the age of the patients (median 25.5 years) [23, 24, 28].

It has been reported that women at reproductive age, taking CHCs, are characterized by a prothrombotic fibrin clot phenotype with prolonged lysis, and that discontinuation of CHCs was associated with shortened CLT and increased *K*_s_ [4]. The current study expands previous findings by comprehensive evaluation of plasma clot structure and function in women with contraception-related VTE. A similarly prothrombic phenotype reflected by reduced *K*_s_ and prolonged CLT was observed in both VTE groups, which is a novel and somewhat intriguing observation. We expected more favorable clot features in those women compared with well-matched comparators after unprovoked VTE. These findings suggest that solely young and middle age, relatively healthy women with a more prothrombotic state could be prone to VTE if they take contraceptives. It might be speculated that there is a subset of women at risk of VTE while taking contraceptives (similarly to those with prior VTE) who could be determined using some coagulation parameters. However, since the variables tested in this study were not measured prior to contraceptives and prior to VTE while on COCs, this hypothesis requires corroboration. Furthermore, lower *K*_s_ with prolonged CLT accompanied by reduced D-D_rate_, that was observed in our study, have been recently demonstrated to predict the VTE recurrence [8, 19]; therefore, it might be speculated that VTE recurrences in women following contraception-related VTE could be at least in part driven by the unfavorable clot properties. It has been shown that subjects with recurrent DVT during follow-up were characterized by slightly lower plasma clot permeability and 15% longer CLT measured at 3 months since the index event compared with the controls [19].

Our study also showed less efficient fibrinolysis in part associated with increased PAI-1:Ag concentration in both VTE groups, which agrees with previous reports [20]. Association of hypofibrinolysis with VTE reflects to some extent the relationship of elevated PAI-1 and the development of VTE [18]. It is known that PAI-1 is positively associated with BMI [15]. In our study, there were no differences in BMI between both VTE groups; therefore, the current intergroup differences in PAI-1 cannot be explained by differences in BMI.

An interesting finding in our study is increased thrombin generation in young and middle-aged women following a few months from the contraception-related VTE group. Interestingly, peak thrombin generated in the contraception-related VTE group was higher to that found in well-matched women following unprovoked VTE. Increased peak thrombin leads to the disturbed balance of pro- and anticoagulant mechanisms during thrombin generation reflected by enhanced prothrombin conversion and reduced thrombin inactivation [29, 30].

Unexpectedly, we observed a higher prevalence of FXIII Val34Leu carriers in women following contraception-related VTE. It is known that this common polymorphism results in the formation of a clot with smaller pores and thinner fibers largely due to faster FXIII activation [31]. Meta-analysis of 11 studies has confirmed that this common FXIII polymorphism can have a moderate protective effect against VTE [32, 33]. Based on the current findings, it might be speculated that the 34Leu FXIII allele could contribute to the risk of contraception-related VTE in part through unfavorable alterations to clot properties enhanced by hormones. The present observation suggesting the role of Val34Leu allele on contraception-related VTE risk warrants further studies.

It has been convincingly shown that contraceptives with levonorgestrel (the second-generation progestogen) carry a 2- to 4-fold increased risk of VTE, which is the lowest among all generations of COCs [34]. The use of the third-generation progestogens, represented by gestoden, desogestrel, and norgestimate, is associated with 3- to 8-fold increased risk of VTE when compared with nonusers [11]. There is evidence that transdermal patches, containing 15-20 *μ*g of ethinylestradiol per day combined with norgestimate, are associated with a 2-fold increased risk of VTE and appear to be safer than oral preparations [34]. Our study showed that in contrast to women with transdermal contraception-related VTE, participants with contraception-related VTE provoked by oral 2^nd^-generation progestogens have more unfavorable clot properties. It might be speculated that women with the “worse” clot properties are prone to develop VTE while using COCs containing-second generation progestogens, and women with less prothrombotic clot features could be to some extent “resistant” to such adverse events compared with other forms of COCs. However, low numbers of patients in the subgroup analysis render these observations preliminary and they should be treated with extreme caution.

Our study had several limitations. First, the sample size was relatively small but the groups were well-matched and the study was adequately powered. Second, fibrin clot parameters were only analyzed once, shortly after thromboembolic event, and it is unknown whether prothrombotic features reported here persisted. Third, some potential modulators of clot properties, for example TAFI and C3 protein, were unmeasured in this study. Subsequently, women who took COCs to decrease the intensity of premenstrual syndrome (PMS), androgenization described as hirsutism or acne and menstrual bleeding disorders which are present in polycystic ovary syndrome associated with increased risk of VTE [35], were also ineligible. Therefore, the results we have reported in our study, cannot be easily extrapolated to such women. Fourth, in both VTE groups there were more FV Leiden carriers as compared to the control group, although this difference was not significant. Moreover, contrary to the controls, in the VTE group, we observed higher frequency of FXIIIVal34Leu which has affected clot formation and clot structure. Finally, long-term follow-up was beyond the scope of the present study so it remains to be established whether the prothrombotic features are risk factors for VTE recurrence in women following contraception-related VTE, but given recent evidence from a cohort study suggesting that the fibrin clot phenotype has a predictive value of recurrent VTE [8].

## 5. Conclusion

Our study showed that women who experienced contraception-related VTE exhibit denser fibrin clot formation and impaired clot lysis associated with increased PAI-1:Ag concentration. It might be considered to prolong anticoagulant therapy in women with contraception-related VTE if they display prothrombotic clot features after 3 months of treatment. Our study showing enhanced thrombin generation, reflected by increased peak thrombin, and impaired fibrinolysis associated with PAI-1 in women following conception-related VTE could have practical implications. The laboratory markers presented here could be of value in identifying the women at higher risk of VTE.

## Figures and Tables

**Table 1:** Characteristics of the studied groups.

Variable	Contraception-related VTE (*n* = 48)	Unprovoked VTE (*n* = 48)	Control group (*n* = 48)	*p* value
Age (years)	32.6 ± 8.0	32.4 ± 6.6	32.9 ± 8.0	0.9
BMI (kg/m^2^)	27.4 ± 2.3	27.2 ± 3.4	25.9 ± 3.1	0.06
Cigarette smoking, *n* (%)	21 (44)	15 (32)	16 (33)	0.4
Family history of VTE, *n* (%)	11 (23)	9 (19)	4 (8)	0.14
Obesity, *n* (%)	11 (23)	10 (21)	5 (10)	0.33
Arterial hypertension, *n* (%)	4 (8)	14 (29)	11 (23)	0.03
Diabetes mellitus, *n* (%)	1 (2)	2 (4)	3 (6)	0.59
Laboratory parameters				
Fibrinogen (g/L)	2.89 (2.48-3.73)	2.90 (2.36-3.290)	2.76 (2.34-3.42)	0.77
INR	0.98 ± 0.08	0.97 ± 0.16	0.97 ± 0.15	0.89
Creatinine (*μ*mol/L)	61.5 ± 10.73	63.5 ± 7.30	63.9 ± 7.05	0.36
Glucose (mmol/L)	4.95 (4.60-5.45)	4.80 (4.45-5.35)	5.20 (4.80-5.85)	0.60
TG (mmol/L)	1.25 (0.77-1.73)	1.31 (0.85-1.85)	1.18 (0.82-1.61)	0.04
TC (mmol/L)	5.12 ± 1.24	5.35 ± 0.98	4.85 ± 0.89	0.07
HDL-C (mmol/L)	1.51 ± 0.47	1.38 ± 0.38	1.38 ± 0.35	0.20
LDL-C (mmol/L)	3.00 ± 0.92	3.32 ± 0.85	2.92 ± 0.71	0.04
hsCRP (mg/L)	1.18 (0.93-1.78)	1.79 (1.15-2.37)	1.51 (0.91-2.25)	0.11
D-dimer (ng/mL)	272 (212-359)	279 (219-341)	247 (210-300)	0.10
Plasminogen (%)	109.5 ± 13.93	107.4 ± 13.94	108.8 ± 15.77	0.78
*α*_2_-Antiplasmin	99.60 ± 10.42	102.38 ± 9.13	103.6 ± 9.09	0.06
Genetic polymorphisms, *n* (%)				
Factor XIII Val34Leu	23 (48)	16 (33)	10 (21)	0.04
*α*-Fibrinogen Thr312Ala	21 (44)	22 (46)	14 (29)	0.30
Factor V Leiden	8 (17)	7 (15)	2 (4)	0.13
Prothrombin 20210A mutation	2 (4)	2 (4)	2 (4)	1.00

Data are shown as mean ± standard deviation, median (interquartile range) or number (percentage). Abbreviations: HDL-C, high-density lipoprotein cholesterol; hsCRP, high-sensitivity C-reactive protein; INR, international normalized ratio; LDL-C, low-density lipoprotein cholesterol; TC, total cholesterol; TG, triglycerides; and VTE, venous thromboembolism.

**Table 2:** Comparison of fibrin clot properties in the contraception-related VTE group regarding type of contraception.

Variable	Type of contraception			*p*
	CHC 2^nd^ (*n* = 16)	CHC 3^rd^ (*n* = 16)	Transdermal (*n* = 16)	
*K*_s_ (10^–9^cm^2^)	6.6 ± 1.1	7.2 ± 1.3	7.3 ± 1.3	0.36
Lag phase (s)	41.5 ± 7.1	42.1 ± 5.8	43.0 ± 7.0	0.90
*Δ*Abs_max_ (405 nm)	0.82 ± 0.1	0.81 ± 0.1	0.75 ± 0.1	0.26
CLT (min)	86 ± 18.1	90 ± 13.8	93.8 ± 27.8	0.63
D-D_rate_ (mg/L/min)	0.068 ± 0.006	0.070 ± 0.004	0.077 ± 0.009	0.007
D-D_max_ (mg/L^A^)	4.0 (3.4-4.1)	3.70 (3.4-4.2)	3.66 (3.3-3.7)	0.05

Data are shown as mean ± standard deviation or median (interquartile range). Abbreviations: CHC, combined hormonal contraception containing estrogen plus 2^nd^- or 3^rd^-generation progesterone; *Δ*Abs_max_, maximum absorbance at the plateau phase; CLT, clot lysis time; D-D_max_, maximum D-dimer levels in the lysis assay; D-D_rate_, maximum rate of increase in D-dimer levels in the lysis assay; *K*_s,_ fibrin clot permeability coefficient.

## Data Availability

(1) All data created during this research is openly available from the Institute of Cardiology, Jagiellonian University Medical College, and John Paul II Hospital, Krakow, Poland. (2) The datasets generated during and/or analyzed during the current study are available from the corresponding author upon reasonable request. (3) All data supporting this study is provided as supplementary information accompanying this paper.

## Additional Points

*Highlights*. Women with contraception-related VTE exhibit denser fibrin clot formation and impaired clot lysis. Prolonged anticoagulant therapy in women with prothrombic clot features after contraception-related VTE should be considered. Enhanced thrombin generation, reflected by increased peak thrombin, and impaired fibrinolysis associated with PAI-1 in women taking combined hormonal contraceptives increased the risk of VTE.

## Supplementary Materials

Supplementary Materials The supplementary materials consist of correlation coefficients for specific coagulation and fibrinolysis markers analyzed during the study. Table S1: correlation coefficients for the permeability coefficient (*K*_s_), maximum D-dimer levels in the lysis assay (D-D_max_), and maximum rate of increase in D-dimer levels in the lysis assay (D-D_rate_).
