# Supplementary material for: Plasma Fibrin Clot Properties Are Unfavorably Altered in Women following Venous Thromboembolism Associated with Combined Hormonal Contraception
Source: Dis Markers. 2019 Nov 18;2019:4923535. doi: 10.1155/2019/4923535 (PMC6885764; doi:10.1155/2019/4923535)
Supplement: Supplementary Materials — The supplementary materials consist of correlation coefficients for specific coagulation and fibrinolysis markers analyzed during the study. Table S1: correlation coefficients for the permeability coefficient (Ks), maximum D-dimer levels in the lysis assay (D-Dmax), and maximum rate of increase in D-dimer levels in the lysis assay (D-Drate). [file 4923535.f1.pdf]

Supplementary Material

Table S1. Correlation coefficients for the permeability coefficient (K_s), maximum D-dimer levels in the lysis assay ($D\text{-}D_{max}$) and maximum rate of increase in D-dimer levels in the lysis assay ($D\text{-}D_{rate}$).

	K_s	$D\text{-}D_{max}$	$D\text{-}D_{rate}$
**Contraception-related VTE**			
Fibrinogen	-0.49	0.67	-0.45
Lag pahse	0.55	-0.38	NC
ΔAbs_{max}	-0.69	0.63	-0.52
**Unprovoked VTE**			
Fibrinogen	-0.54	0.52	NC
Lag pahse	NC	-0.40	NC
ΔAbs_{max}	-0.41	0.45	NC
**Controls**			
Fibrinogen	-0.64	0.68	NC

Lag pahse	NC	NC	NC
ΔAbs_{max}	-0.52	-0.43	NC

Abbreviations: ΔAbs_{max}, maximum absorbance at the plateau phase; CLT, clot lysis time; D-D_{max}, maximum D-dimer levels in the lysis assay; D-D_{rate}, maximum rate of increase in D-dimer levels in the lysis assay; $K_{s,}$ fibrin clot permeability coefficient; NC indicates nonsignificant correlation.
